# Analysis of Meiosis in SUN1 Deficient Mice Reveals a Distinct Role of SUN2 in Mammalian Meiotic LINC Complex Formation and Function

**DOI:** 10.1371/journal.pgen.1004099

**Published:** 2014-02-27

**Authors:** Jana Link, Monika Leubner, Johannes Schmitt, Eva Göb, Ricardo Benavente, Kuan-Teh Jeang, Rener Xu, Manfred Alsheimer

**Affiliations:** 1 Department of Cell and Developmental Biology, Biocenter, University of Würzburg, Würzburg, Germany; 2 Molecular Virology Section, Laboratory of Molecular Microbiology, National Institute of Allergy and Infectious Diseases, National Institutes of Health, Bethesda, Maryland, United States of America; 3 Institute of Developmental Biology and Molecular Medicine and School of Life Science, Fudan University, Shanghai, China; Max F. Perutz Laboratories, University of Vienna, Austria

## Abstract

LINC complexes are evolutionarily conserved nuclear envelope bridges, composed of SUN (Sad-1/UNC-84) and KASH (Klarsicht/ANC-1/Syne/homology) domain proteins. They are crucial for nuclear positioning and nuclear shape determination, and also mediate nuclear envelope (NE) attachment of meiotic telomeres, essential for driving homolog synapsis and recombination. In mice, SUN1 and SUN2 are the only SUN domain proteins expressed during meiosis, sharing their localization with meiosis-specific KASH5. Recent studies have shown that loss of SUN1 severely interferes with meiotic processes. Absence of SUN1 provokes defective telomere attachment and causes infertility. Here, we report that meiotic telomere attachment is not entirely lost in mice deficient for SUN1, but numerous telomeres are still attached to the NE through SUN2/KASH5-LINC complexes. In *Sun1^−/−^* meiocytes attached telomeres retained the capacity to form bouquet-like clusters. Furthermore, we could detect significant numbers of late meiotic recombination events in *Sun1^−/−^* mice. Together, this indicates that even in the absence of SUN1 telomere attachment and their movement within the nuclear envelope *per se* can be functional.

## Author Summary

Correct genome haploidization during meiosis requires tightly regulated chromosome movements that follow a highly conserved choreography during prophase I. Errors in these movements cause subsequent meiotic defects, which typically lead to infertility. At the beginning of meiotic prophase, chromosome ends are tethered to the nuclear envelope (NE). This attachment of telomeres appears to be mediated by well-conserved membrane spanning protein complexes within the NE (LINC complexes). In mouse meiosis, the two main LINC components SUN1 and SUN2 were independently described to localize at the sites of telomere attachment. While SUN1 has been demonstrated to be critical for meiotic telomere attachment, the precise role of SUN2 in this context, however, has been discussed controversially in the field. Our current study was targeted to determine the factual capacity of SUN2 in telomere attachment and chromosome movements in SUN1 deficient mice. Remarkably, although telomere attachment is impaired in the absence of SUN1, we could find a yet undescribed SUN1-independent telomere attachment, which presumably is mediated by SUN2 and KASH5. This SUN2 mediated telomere attachment is stable throughout prophase I and functional in moving telomeres within the NE. Thus, our results clearly indicate that SUN1 and SUN2, at least partially, fulfill redundant meiotic functions.

## Introduction

Nuclear anchorage and movement, including the directed repositioning of components within the nucleus, are essential for coordinated cell division, proliferation and development [1]. As these processes are largely dependent on cytoskeletal components, the cytoskeleton needs to interact with both the nuclear envelope (NE) and the nuclear content [2]. In this context, the so-called LINC (linker of nucleoskeleton and cytoskeleton) complexes emerged as the key players in that they represent the central connectors of the nucleus and its content to diverse elements of the cytoskeleton [2]–[4]. LINC complexes are widely conserved in evolution regarding their composition and function. They are composed of SUN (Sad-1/UNC-81) domain proteins that reside in the inner nuclear membrane (INM) which bind to KASH (Klarsicht/ANC-1/Syne/homology) domain proteins of the outer nuclear membrane (ONM) [4], [5]. Through specific interactions of SUN domain proteins with nuclear components, such as lamins, and the interactions of KASH domain proteins with the cytoskeleton, the SUN-KASH complexes are able to transfer mechanical forces of the cytoskeleton directly to the NE and into the nucleus [6], [7].

During meiosis, telomeres are tethered to and actively repositioned within the NE. The characteristic telomere-led chromosome movements are an evolutionarily highly conserved hallmark of meiotic prophase I; they are a prerequisite for ordered pairing and synapsis of homologous chromosomes [8], [9]. Directed chromosome movement, pairing and recombination are closely interdependent processes and their correct progression is essential for the faithful segregation of homologous chromosomes into fertile gametes. Failure in any of these processes leads to massive meiotic defects and, consistent with this, mutant mice showing defects in meiotic telomere attachment, chromosome dynamics or synapsis formation are mostly infertile due to apoptosis during prophase I [10]–[13].

The attachment of meiotic telomeres to the NE is mediated by SUN-KASH protein complexes [11], [14]–[21]. Of the five SUN-domain proteins known in mammals, SUN1 and SUN2 have been shown to be the only ones that are also expressed in meiotic cells [11], [22]. Recently, a novel meiosis-specific KASH domain protein, KASH5, has been identified as a constituent of the meiotic telomere attachment complex [23], [24]. With this, the first fully functional and complete mammalian meiotic LINC complex comprised of SUN1 and/or SUN2 within the INM and KASH5 as the ONM partner has been characterized. Nonetheless, many aspects of mammalian meiotic telomere attachment and movement, including its regulation, are not yet fully understood. To date, SUN1 and SUN1/SUN2 deficient mice have been studied to investigate both somatic and meiotic functions of SUN1 and SUN2 [11], [21], [25], [26]. These studies have provided clear evidence that in somatic cells SUN1 and SUN2 play partially redundant roles. However, it also turned out that mice deficient in SUN1 are infertile due to serious problems in attaching meiotic telomeres to the nuclear envelope [11], [21], demonstrating the importance of SUN1 for meiotic cell division. Although SUN2 was found to be present at the sites of telomere attachment during meiotic prophase I, the SUN1 deficient phenotype demonstrated that SUN2 apparently is not able to effectively compensate for the loss of SUN1 in meiosis [11], [21], [22]. To learn more about the distinctive roles of SUN1 and SUN2 in meiotic telomere function and behavior we started a detailed re-evaluation of the meiotic phenotype caused by SUN1 deficiency. In our current study we now show that in the absence of SUN1 meiotic telomere attachment actually is not entirely lost, pointing to the existence of a SUN1-independent, partially redundant attachment mechanism. Consistent with this, we could find that in *Sun1^−/−^* mice NE-attached telomeres co-localize with SUN2 and KASH5, suggesting that telomere attachment is mediated by SUN2/KASH5-LINC complexes in SUN1 deficient meiocytes. Furthermore, *Sun1^−/−^* meiocytes showed clustering patterns of the NE-attached telomeres that resembled typical bouquet-like configurations, indicating that SUN2 is not only sufficient to connect a significant portion of telomeres to the NE, but rather is part of a functional LINC complex capable of transferring cytoplasmic forces required to move telomeres.

## Results and Discussion

### Though NE-attachment of telomeres is disturbed in SUN1 deficient mice, numerous telomeres can still be found attached to the NE

**Figure 1:**
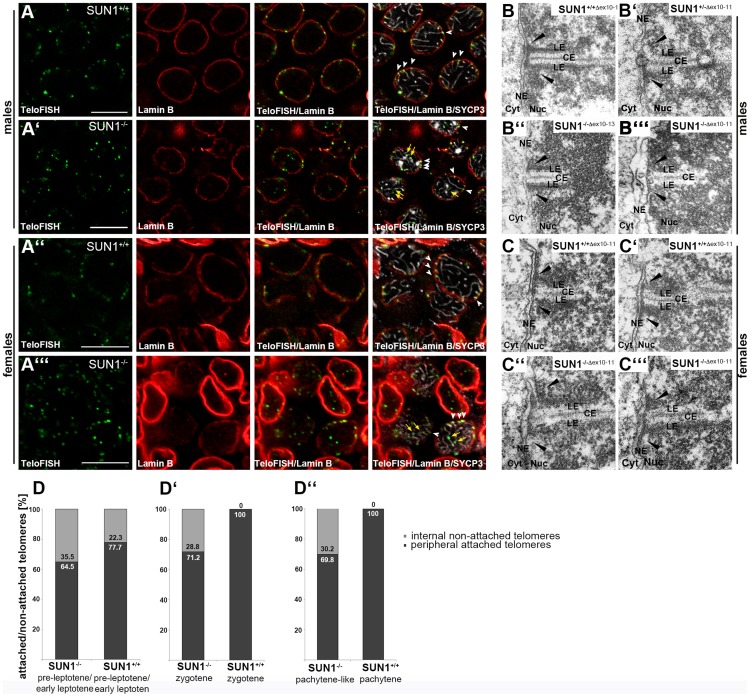
Presence of meiotic telomere attachment in *Sun1^−/−^* mouse strains. (A) Telomere fluorescence in-situ hybridization (TeloFISH) in co-localization with Lamin B and SYCP3 immunofluorescence on representative 15 dpp (days past partum) *Sun1^+/+(Δex10-11)^* and littermate *Sun1^−/−(Δex10-11)^* testis sections (A, A′) and 17.5 dpf (days past fertilization) *Sun1^+/+(Δex10-11)^* and littermate *Sun1^−/−(Δex10-11)^* ovary sections (A″, A′″). In all WT sections investigated, attached telomeres appear embedded within the labeled lamina (white arrowheads in A and A″). All sections from knockout tissues clearly show both detached, internal telomere signals (yellow arrows in A′ and A′″) as well as attached, peripheral telomere signals (white arrowheads in A′ and A′″) in both oocytes and spermatocytes. Peripheral, attached telomeres in SUN1 deficient oocytes and spermatocytes are also seen at the ends of synaptonemal complex (SC) axes shown by SYCP3, as is the case in wildtype cells. Scale bar 10 µm. (B) Representative electron micrographs of spermatocytes from adult *Sun1^+/+(Δex10-13)^* (B) and *Sun1^−/−(Δex10-13)^* (B″) [11] mice and of spermatocytes from 15 dpp *Sun1^+/−(Δex10-11)^* (B′) and *Sun1^−/−(Δex10-11)^* (B′″) [21] mice. (C) Representative electron micrographs from E17.5 female *Sun1^+/+(Δ10-11)^* (C–C′) and *Sun1^−/−(Δ10-11)^*(C″–C′″) oocytes. In male wildtype meiocytes of both mouse strains and female wildtype meiocytes of the *SUN1^(Δex10-11)^* strain, components of the SC and the telomere attachment plates (black arrowheads) are clearly visible. Meiocytes from all *Sun1^−/−^* males (B″– B′″) as well as females (C″–C′″) also show the wildtype-like formation of telomere attachment sites. (D) Quantification of attached and non-attached telomeres in wildtype and knockout spermatocytes at different meiotic stages. Pre-leptotene/early leptotene spermatocytes from littermate 12 dpp mice (D), zygotene spermatocytes from littermate 12 dpp mice (D′) and spermatocytes from littermate 14 dpp mice in a pachytene or pachytene-like stage, respectively (D″). (12 dpp pre-leptotene/early leptotene: *Sun1^+/+(Δex10-11)^ n* = 16 spermatocytes, 772 telomeres; *Sun1^−/−(Δex10-11)^ n* = 13 spermatocytes, 645 telomeres. 12 dpp zygotene: *Sun1^+/+(Δex10-11)^ n* = 5 spermatocytes, 194 telomeres; *Sun1^−/−(Δex10-11)^ n* = 7 spermatocytes, 337 telomeres. 14 dpp pachytene: *Sun1^+/+(Δex10-1)1^ n* = 54 spermatocytes, 2138 telomeres; pachytene-like *Sun1^−/−(Δex10-11)^ n* = 31 spermatocytes, 1150 telomeres.) LE lateral element, CE central element, NE nuclear envelope, Nuc nucleoplasm, Cyt Cytoplasm.

In recent years, it has been established by several groups that meiotic telomere attachment in mammals involves SUN1 and SUN2 as part of the NE spanning LINC complex connecting the meiotic telomeres to the cytoskeleton [11], [21], [22]. To analyze SUN1 function, two independent SUN1 deficient mouse models have been generated so far (here referred to as *Sun1^(Δex10-13)^* [11] and *Sun1^(Δex10-11)^* [21]), which both revealed a virtually identical, exclusively meiotic phenotype: both male and female SUN1 deficient mice showed severe meiotic defects, which were ascribed to massive problems in meiotic telomere attachment [11], [21]. Although SUN2 compensates for the loss of SUN1 in somatic cells, SUN2 overtly does not have the competence to counterbalance loss of SUN1 in meiocytes, and hence it was described that telomere attachment is prevented in *Sun1^−/−(Δex10-13)^* mice [11]. Since we have previously found SUN2 expressed in meiocytes, where it localizes to the sites of telomere attachment [22], this raises the question of the real function of SUN2 in meiosis. To investigate the actual role of SUN2 during meiosis, we therefore initiated a detailed analysis of telomere attachment in SUN1 deficient meiocytes and started off with spermatocytes and oocytes from *Sun1^−/−(Δex10-11)^* mice, which were previously demonstrated to be SUN1 deficient [21]. Worth mentioning, using antibodies recognizing an epitope encoded by exons 13 to 14 [27] we could confim that these mice in fact do not express a functional SUN1 protein (data not shown). To study telomere behavior in SUN1 deficient mice, we combined telomere fluorescence in-situ hybridization with immunocytochemical labeling of the lamina and the synaptonemal complexes in spermatocytes and oocytes of SUN1 knockout and wildtype littermate mice (Figure 1A). As expected, in wildtype spermatocytes and oocytes all telomere signals that are clearly associated with the ends of synaptonemal complexes, are embedded within the lamina (Figure 1A and A″). Consistent with the previously published results [11], [21], we found that telomere attachment to the nuclear envelope is significantly disturbed in SUN1 deficient meiocytes (Figure 1A′ and A′″). This is evident from telomere signals located in the nuclear interior, in significant distance to the NE. However, within the same meiocytes, we found that numerous telomere signals were still embedded within the lamina (arrowheads in A′ and A′″), indicating that in the absence of SUN1 telomere attachment may not be entirely lost, but only reduced. The unexpected high numbers of peripheral, nuclear envelope associated telomere signals that were observed in both spermatocytes and oocytes of *Sun1^−/−(Δex10-11)^* mice (see below) gave the impression that at least a portion of the peripheral telomeres might be structurally anchored at the nuclear envelope, which would clearly contradict the previous notion that loss of SUN1 completely prevents telomere attachment [11]. To clarify whether these telomeres are truly attached or merely located in close vicinity to the NE, we therefore prepared testis tissue and ovary samples for electron microscopy, as both synaptonemal complexes and sites of telomere attachment can easily be detected in electron micrographs (Figure 1B–B′, C–C′). To affirm that putative attachment does not depend on the knockout genotype, we analyzed samples from both currently available SUN1 deficient strains, *Sun1^(Δex10-13)^* [11] and *Sun1^(Δex10-11)^* [21]. As anticipated, fully synapsed stretches of synaptonemal complexes attached to the nuclear envelope were clearly evident in all control samples of pachytene spermatocytes and oocytes. Remarkably, oocytes and spermatocytes from both SUN1 deficient mouse strains revealed similar telomere attachment sites to the ones observed in the wildtype (Figure 1B″–B′″, C″–C′″). Although many homologous chromosomes in both *Sun1^−/−^* mice strains fail to pair and synapse during pachynema [11], [21], partially completed synaptonemal complexes are still present in pachytene-like staged meiocytes. When these are tethered to the NE, wildtype-like attachment sites seem to be able to form. Together, the immunocytochemical (see below) and electron micrograph data show that telomere attachment is not completely abolished during meiosis in mice lacking SUN1, irrespective of the genetic targeting strategy used to create the SUN1 deficient mouse strain. Together, our findings presented here in fact proved that even in the absence of SUN1 a subset of meiotic telomeres is still able to attach to the NE, and thus our results refute the previous assumption regarding the lack of telomere attachment in SUN1 deficient mice [11]. Particularly the use of electron microscopic analysis on SUN1 deficient meiocytes has revealed some of the phenotypic features, which have been overtly overlooked before.

To define the percentage of attached telomeres in *Sun1^−/−(Δex10-11)^* spermatocytes we quantified the number of attached and non-attached telomeres in 3 dimensionally preserved nuclei of cells, simultaneously labeled for the nuclear lamina, the synaptonemal complexes and telomeres. To evaluate further whether the absence of SUN1 impacts telomere attachment in a stage dependent manner during meiotic progression, we additionally quantified and compared telomere attachment in spermatocytes at early leptonema, zygonema and at pachynema. For this we prepared tissue samples of wildtype and knockout littermates aged 12 and 14 days post partum (dpp). As in the first wave of spermatogenesis development of spermatocytes within the seminiferous tubules is nearly synchronized [28], at 12 dpp most spermatocytes within the tubules could be found at early leptonema to early zygonema. In tubules where early leptotene spermatocytes predominated, telomere attachment was not complete in both wildtype and knockout spermatocytes, probably due to the very early meiotic stage (77.7% and 64.5% attached telomeres in wildtype and knockout, respectively; Figure 1 D; Figure S1). In tubules where early zygotene spermatocytes were accumulated all wildtype spermatocytes showed complete telomere attachment, whereas in knockout zygotene spermatocytes not more than 71.2% of all telomeres appeared to be NE-attached (Figure 1 D′; Figure S1). We observed similar rates of telomere attachment in spermatocytes of 14 dpp mice, where pachytene stages predominated. Here, wildtype spermatocytes again showed complete attachment of all telomeres, whereas *Sun1^−/−(Δex10-11)^* males only showed 69.8% of telomeres attached to the NE (Figure 1 D″). These results implicate that the process of telomere attachment is induced despite SUN1 deficiency, yet full telomere attachment is never reached. Almost equivalent rates of attachment could be detected in zygotene and pachytene spermatocytes of *Sun1^−/−(Δex10-11)^* mice, suggesting that once telomeres succeed to attach they maintain their association with the NE throughout prophase I, even in the absence of SUN1. This indicates that attachment of telomeres to the NE without SUN1 is stable enough to withstand potential mechanical forces generated by the chromatin or cytoskeleton. The unexpected, relatively large proportion of telomeres that, without SUN1, are still capable of stably attaching to the NE clearly points towards the existence of a partially redundant and SUN1-independent attachment mechanism.

### KASH5 localizes to NE-associated telomeres in SUN1 deficient meiocytes

**Figure 2:**
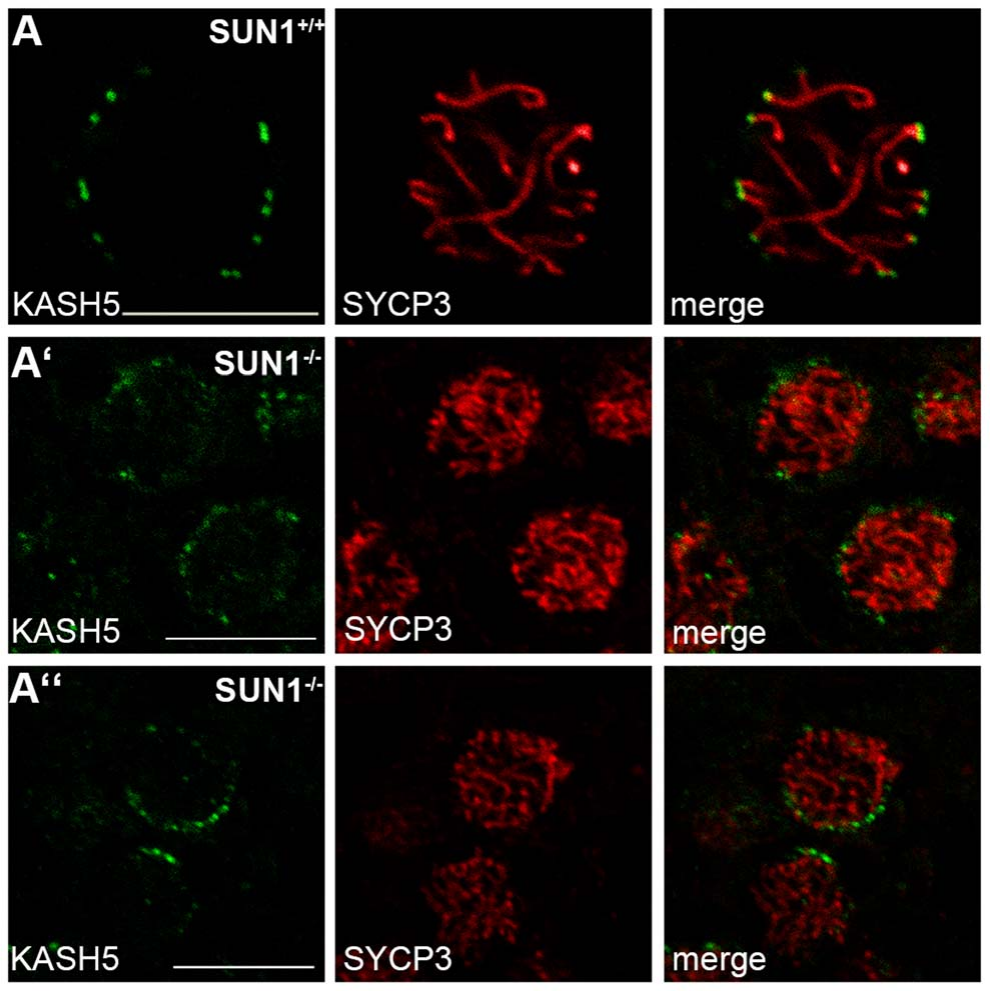
KASH5 localization in SUN1 deficient males. Representative spermatocytes in paraffin sections of *Sun1^+/+(Δex10-11)^* and *Sun1^−/−(Δex10-11)^* testis stained for SYCP3 and KASH5. In the wildtype (A) the expected KASH5 localization at the distal ends of synaptonemal complex axes can clearly be observed. In *Sun1^−/−(Δex10-11)^* spermatocytes (A′–A″) the KASH5 signal, although weaker, is also clearly detectable. As seen in the wildtype, distinct KASH5 foci also co-localize with the ends of synaptonemal complex axes. Scale bars 10 µm.

Very recently, it has been described that meiotic tethering of telomeres to the cytoskeleton is mediated by the novel meiosis-specific KASH-protein KASH5 [23], [24]. To clarify whether KASH5 is also involved in the attachment of telomeres in SUN1 deficient meiocytes, we conducted immunofluorescence experiments labeling KASH5 and SYCP3, a major component of the lateral elements of synaptonemal complexes [29], in wildtype and SUN1 knockout spermatocytes. Consistent with earlier reports [23], [24], strong KASH5 foci at the ends of synaptonemal complexes were detected in all wildtype pachytene spermatocytes (Figure 2 A), labeling telomeres attached to the NE. However, in contradiction to earlier reports [23], [24], in our hands KASH5 foci were also consistently present in *Sun1^−/−(Δex10-11)^* spermatocytes in several independent experiments and different animals tested (Figure 2 A′, A″). Although significantly weaker than in the wildtype tissue, the KASH5 signals in SUN1 deficient meiocytes nevertheless showed a wildtype-like distribution. KASH5 in the SUN1 deficient spermatocytes was found to be localized just at those ends of synaptonemal complexes that are in close contact with the NE. These experiments again corroborate that in the absence of SUN1 the remaining NE-associated telomeres are indeed attached to the NE. Beyond this, the attached telomeres are connected to the cytoskeleton through a linkage that involves KASH5.

### Even in the absence of SUN1, SUN2 co-localizes with KASH5 at the sites of telomere attachment

**Figure 3:**
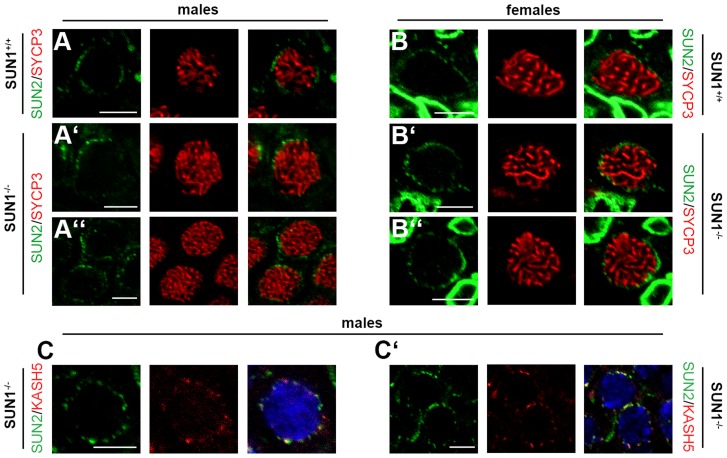
Meiotic telomere tethering by LINC complex components in the absence of SUN1. (A,B) Representative meiocytes in paraffin sections of testis and ovary tissue of *Sun1^+/+(Δex10-11)^* and *Sun1^−/−(Δex10-11)^* mice labeled by anti-SUN2 and anti-SYCP3 antibodies. SUN2 foci, located at the end of synaptonemal complex axes, are present in both wildtype spermatocytes and oocytes (A, B). Similar SUN2 signals are also present in spermatocytes and oocytes of SUN1 deficient littermate mice (A′, A″, B′, B″). The nuclear envelope of somatic cells in the ovary tissue of both *Sun1^+/+(Δex10-11)^* and *Sun1^−/−(Δex10-11)^* females (B–B″) is also strongly labeled by SUN2. (C–C′) Spermatocytes in paraffin sections of testis tissue of *Sun1^−/−(Δex10-11)^* males labeled by anti-SUN2 and anti-KASH5 antibodies. In SUN1 deficient spermatocytes KASH5 and SUN2 co-localize, both showing distinct foci at the nuclear periphery. DNA counterstained using Hoechst 33258. Scale bars 5 µm.

In an earlier publication [22] we were able to demonstrate that SUN2 is expressed throughout meiotic prophase I, where it co-localizes with attached telomeres in wildtype mice. Therefore, it is tempting to speculate that telomere attachment in the absence of SUN1 is mediated by SUN2. To follow up on this, we generated SUN2 specific antibodies and used these in co-immunolocalisation experiments together with antibodies against SYCP3. Consistent with our previous results, our newly generated antibodies produced the already reported SUN2 foci at the end of synaptonemal complex axes in both wildtype spermatocytes and oocytes (Figure 3 A, B; [22]). Similar to the wildtype situation, SUN2 foci of comparable intensities were also present in spermatocytes and oocytes of different meiotic prophase stages from *Sun1^−/−(Δex10-11)^* mice (Figure 3 A′, A″, B′, B″). This again demonstrates that SUN2 is indeed located at meiotic telomeres. As SUN2 is the only SUN domain protein expressed in *Sun1^−/−^* meiocytes, it appears likely that it is in fact SUN2 that mediates the observed telomere attachment in the SUN1 deficient mice. To further investigate attachment of telomeres in the *Sun1^−/−(Δex10-11)^* mice, in particular with regard to possible KASH protein partners, we conducted co-immunostaining experiments using KASH5 and SUN2 antibodies on paraffin testis sections from mice of different ages (12 dpp and adult) (Figure 3 C, C′). Clearly, as anticipated for a functional meiotic LINC-complex, the KASH5 and SUN2 foci in the *Sun1^−/−(Δex10-11)^* spermatocytes co-localized, labeling those telomeres that are attached to the NE in the absence of SUN1. In summary, these results indicate that the SUN2 localization to meiotic telomeres can occur independently of SUN1, which is in accordance with the previous reports of unchanged SUN2 localization in somatic nuclei of *Sun1^−/−^* mice [26]. Furthermore, by means of the results presented here, SUN2 appears to be, at least to some extent, sufficient for meiotic telomere attachment to the NE. Regarding its possible interaction with KASH5, yeast-two-hybrid studies have previously shown that the KASH domain of KASH5 in effect is able to interact with both the C-terminal domain of SUN1 as well as of SUN2 [23]. This, in combination with our results, leads us to the conclusion that SUN2 may also form functional meiotic LINC complexes with KASH5 *in vivo*, which, at least in the absence of SUN1, is able to tether meiotic telomeres to the NE.

In a recent crystallography study investigating LINC complex structure, SUN and KASH domains were shown to interact as two sets of trimeric protein complexes [30]. Furthermore, several groups have proposed SUN1 and SUN2 to form hetero-multimeric complexes [31], [32]. Taking into account that SUN2 is expressed during meiosis (present study, [22]), sharing its localization with SUN1 and KASH5, it is tempting to speculate that during wildtype meiosis SUN1 and SUN2 assembly heterotrimeric complexes that interact with KASH5 to form meiotic LINC complexes required for efficiently tethering telomeres to the NE. In the absence of SUN1, such LINC complexes may only be composed of SUN2 and KASH5, still tethering telomeres to the NE, yet in a less effective manner than a complete heterotrimeric SUN1/SUN2- KASH5 complex. This could then explain the only partially disturbed telomere attachment observed in both SUN1 deficient mouse models. In addition, our results presented here suggest at least partial redundancy between SUN1 and SUN2 in meiotic telomere attachment, consistent with what has been reported for nuclear anchorage in somatic cells [25], [26].

### NE-attached telomeres are still capable of forming bouquet-like clusters in SUN1 deficient meiocytes

**Figure 4:**
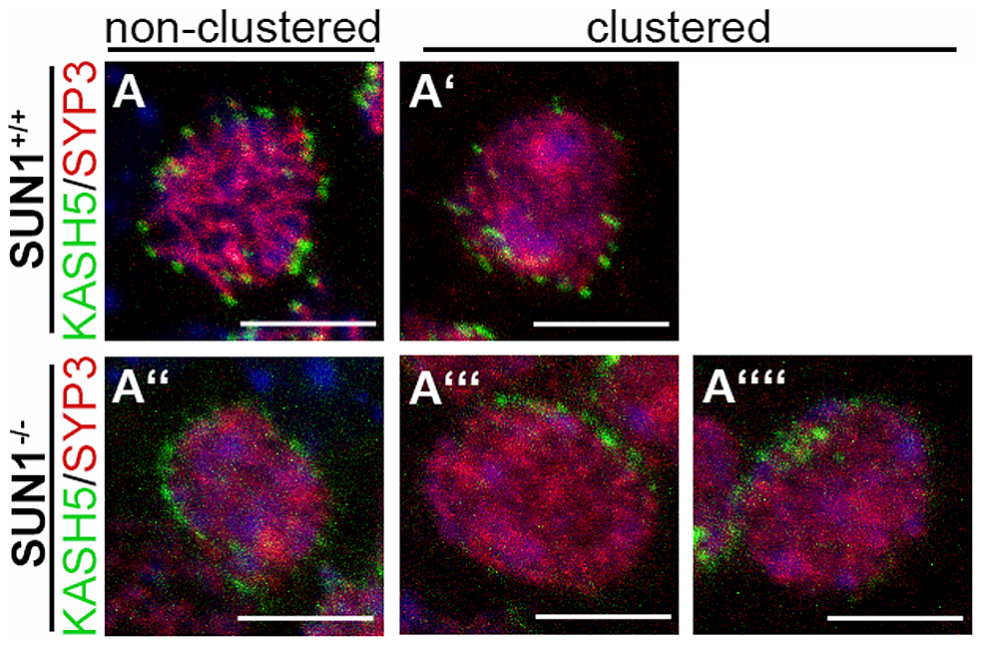
Meiotic telomere clustering in the absence of SUN1. Representative projections of entire spermatocyte nuclei of *Sun1^+/+(Δex10-11)^* and *Sun1^−/−(Δex10-11)^* mice labeled by KASH5 and SYCP3. As expected non-clustered (A) and clustered (A′) telomere patterns are observed in wildtype spermatocytes. Similar non-clustered (A″) as well as clustered (A′″–A″″) telomere patterns could also be found in SUN1 deficient spermatocytes. All scale bars 5 µm.

Prophase I of meiosis is not only characterized by the stable association of telomeres with the NE, but also by directed telomere-led chromatin movements leading to the formation and release of the bouquet stage [8]. Because SUN1 seems to be, at least partially, dispensable for the formation of a meiotic LINC complex *per se*, we asked whether those telomeres, which attach to the NE despite the absence of SUN1, are still able to move along and to cluster within the NE. To analyze the distribution of the attached telomeres in the *Sun1^−/−(Δex10-11)^* mice, we used KASH5 and SYCP3 antibodies for labeling attached telomeres in relation to synaptonemal complexes in spermatocytes of wildtype and knockout siblings at 12 dpp (Figure 4). At this age, leptotene/zygotene stages showing clustered telomere patterns normally predominate within the synchronously maturing tubules. To define KASH5 distribution within the NE, we performed 3D reconstructions of single spermatocyte nuclei of wildtype (*n* = 50 cells) and knockout (*n* = 64 cells) mice. Spermatocytes showing typically clustered KASH5 patterns resembling bouquet-like conformations of the attached telomeres could be detected in both wildtype and SUN1 knockout siblings (Figure 4 and Supplementary Video S1). Further quantifications with respect to the appearance of clustered versus non-clustered KASH5 patterns revealed that at 12 dpp bouquet frequencies were similar and statistically indifferent between wildtype and *Sun1^−/−(Δex10-11)^* siblings (70% and 79.6%, respectively; *p-value* 0.23 Pearson's chi square test). These analyses demonstrated that the remaining attached telomeres in SUN1 deficient males in fact are able to form bouquet-like clustered telomere patterns and that this is not a rare event but occurs at similar rates as in the wildtype siblings. It is noteworthy, that we never observed a real clustering of the internal non-attached telomeres in Sun1 deficient spermatocytes. Taken together, we conclude from this that telomeres need to be attached to the NE, likely connected to the cytoskeleton, to form bouquet-like clusters. In *Smc1ß^−/−^* mice [33], another knockout mouse model where telomere attachment is partially disrupted, bouquet formation of attached telomeres was observed in knockout spermatocytes as well, although at reduced levels compared to the wildtype. Regarding this study and our results, it seems conceivable that completed telomere attachment *per se* is not an essential prerequisite for telomere clustering. Rather, any telomere which is attached to the NE by a LINC complex has the competence to move within the NE and to proceed to cluster formation.

### A subset of chromosomes from SUN1 deficient oocytes proceeds to cross-over formation

**Figure 5:**
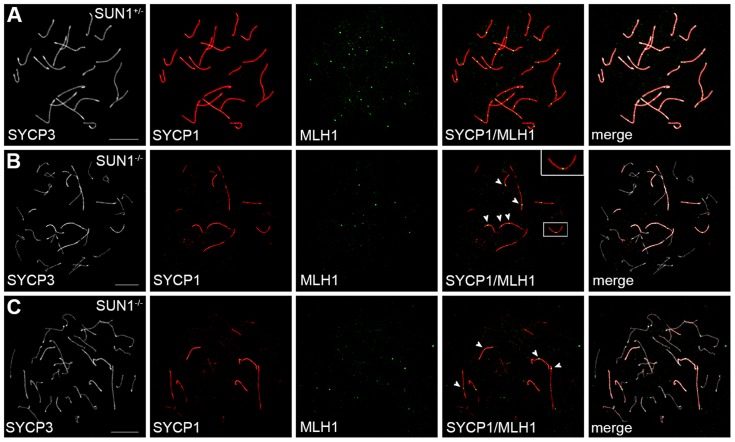
Meiotic recombination in SUN1 deficient oocytes. Representative chromosome spreads of oocytes from 19.5*Sun1^+/+(Δex10-11)^* and *Sun1^−/−(Δex10-11)^* females labeled with anti-SYCP3, anti-SYCP1 and anti-MLH1 antibodies. Complete pairing of all homologous chromosomes as judged by the co-localization of SYCP3 and SYCP1 is observed in heterozygous control pachytene oocytes (A). As expected the homolog pairs exhibit 1–2 distinct MLH1 foci each. In SUN1 deficient pachytene-like oocytes (B, C) only some chromosome stretches and few homologous chromosomes are fully paired. Frequent defects in synapsis formation and many univalent chromosomes can be detected, labeled only by SYCP3. However, distinct MLH1 foci can be observed where SYCP3 and SYCP1 co-localize, (arrowheads in B and C). See also inset in B; magnified by a factor of 2. Scale bars 10 µm.

To investigate the impact of the residual telomere attachment and movement on progression of meiotic recombination events, we started a next series of experiments to analyze oocytes of wildtype and *Sun1^−/−(Δex10-11)^* female mice aged 19.5 dpf (days post fertilization) for the appearance of late recombination events. Using antibodies against MLH1, SYCP1 and SYCP3 together on chromosome spreads allowed us to simultaneously investigate late recombination events and the state of synapsis formation. As expected, we observed the expected one to two MLH1 foci per each synapsed chromosome pair on chromosome spreads of the heterozygous control oocytes (Figure 5 A). Consistent with previous reports [11], [21], oocyte spreads from littermate *Sun1^−/−(Δex10-11)^* mice (Figure 5B, C) showed large numbers of unpaired or incorrectly paired chromosome axes stained by SYCP3, but not by SYCP1. Despite these severe synapsis defects, MLH1 foci were not completely absent from *Sun1^−/−(Δex10-11)^* oocyte spreads. Instead, a small number of homologous chromosomes in *Sun1^−/−(Δex10-11)^* oocytes were apparently able to achieve intact synapsis as shown by the complete co-localization of SYCP1 and SYCP3. Distinct MLH1 foci on these fully paired homologs show that they in effect were able to recruit MLH1 to their axis, thus forming cross-over sites. These results indicate that in the absence of SUN1, the remaining attached telomeres and their directed movements within the NE are sufficient to allow at least partial pairing, synapsis and cross-over formation during later meiosis in females. Therefore, when attachment is effectually reached, this attachment *per se* and the following movement of the attached telomeres appear to be functional, at least to some extent, even without SUN1.

In conclusion, from our current study it has become evident, that although SUN1 is essential for the efficient attachment of telomeres to the NE, SUN2 also appears to be involved in the tethering of meiotic telomeres to the NE. In the absence of SUN1, an unexpectedly large proportion of telomeres are still able to attach to the NE and, beyond this, are also able to move within the NE, forming bouquet-like clustered telomere patterns. This suggests that in the SUN1 deficient background some of the telomeres not only succeed to establish a tight connection to the NE, but even become linked to the cytoskeletal motor system. Consistent with this, in the SUN1 deficient meiocytes we found KASH5, which interacts with cytoplasmic dynein–dynactin [23], [24], co-localizing with SUN2 at sites where telomeres are in contact with the NE. In a very recent study, Horn and colleagues [24] have shown that in mice deficient for KASH5, homolog pairing, synapsis and recombination is severely disturbed. In addition, they never observed clustering of SUN1 foci in KASH5 deficient cells, indicating that KASH5 as the ONM component of meiotic LINC complexes is required for transferring forces to move the INM located SUN proteins and therewith the attached telomeres [24]. Remarkably, the meiotic phenotype observed in the *Kash5*-null mice appeared much more dramatic than the phenotype induced by SUN1 deficiency. As shown by Horn and colleagues *Kash5*-null spermatocytes overtly never reach full synapsis not even of single pairs of homologous chromosomes, while in a considerable proportion of *Sun1*-null spermatocytes full synaptic pairing of at least a subset of homologs could be observed [11], [24]. This is consistent with our results demonstrating that attached telomeres in SUN1 deficient mice in effect are able to cluster, most likely mediated by a restricted LINC complex formed by KASH5 and SUN2, hence supporting synapsis and recombination. To date, no mammalian model has been described where meiotic telomere attachment is completely lost. Instead there are a number of phenotypes with more or less severe partial telomere attachment defects, similar to the *Sun1^−/−^* phenotype described here [33], [34]. This is unlike the situation in yeast, for example, where bqt4 has been identified as a key player without which no meiotic telomeres attach to the NE at all [15]. The meiotic telomere attachment in mammals, however, seems to be regulated by a more complex, partially redundant network of factors, of which some of the central players await identification in the near future.

## Materials and Methods

### Ethics statement

All animal care and experiments were conducted in accordance with the guidelines provided by the German Animal Welfare Act (German Ministry of Agriculture, Health and Economic Cooperation). Animal housing and breeding at the University of Würzburg was approved by the regulatory agency of the city of Würzburg (Reference ABD/OA/Tr; according to §11/1 No. 1 of the German Animal Welfare Act). All aspects of the mouse work were carried out following strict guidelines to ensure careful, consistent and ethical handling of mice.

### Animals and tissue preparations

Tissues used in this study were derived from wildtype, heterozygous and knockout littermates of either of the two currently existing SUN1 deficient mouse strains, *Sun1^(Δex10-13)^* and *Sun1^(Δex10-11)^* [11], [21]. For immunofluorescence studies testes and ovaries from wildtype, heterozygous and SUN1 knockout progeny of the *Sun1^(Δex10-11)^* strain were fixed for 3 hrs in 1% PBS-buffered formaldehyde (pH 7.4). Tissues were then dehydrated in an increasing ethanol series, infiltrated with paraffin wax at 58°C overnight and embedded in fresh paraffin wax as described in Link et al. [13]. For EM analysis we prepared tissue material from wildtype, heterozygous and SUN1 deficient mice from both SUN1 deficient mouse strains, the *Sun1^(Δex10-13)^* and *Sun1^(Δex10-11)^* strain, according to the protocol described below.

### Antibodies

For the generation of SUN2 specific antibodies, a His-tagged SUN2 fusion construct (amino acids 248–469 of the SUN2 protein) was expressed in *E. coli* RosettaBlue (Novagen, Darmstadt, Germany) and purified through Ni-NTA agarose columns (Qiagen, Düsseldorf, Germany). This peptide was used for immunization of a guinea pig (Seqlab, Göttingen, Germany). The serum obtained was affinity purified against the SUN2 antigen coupled to a HiTrap NHS-activated HP column (GE Healthcare, Munich, Germany). Similarly, for the generation of a KASH5 specific antibody, a His-tagged KASH5-fusion construct (amino acids 421–612) was expressed and purified as described above. This peptide was used for immunization of a rabbit and the serum obtained was purified using a KASH5 antigen coupled HiTrap NHS-activated HP column. Further primary antibodies used in this study were: goat anti-Lamin B antibody (Santa Cruz Biotechnology, Heidelberg, Germany), rabbit anti-SYCP3 antibody (anti-Scp3; Novus Biologicals, Littleton, CO), guinea pig anti-SUN1 antibody [27] and mouse anti-KASH5 [23]. For TeloFISH analyses we further used monoclonal mouse anti-digoxigenin antibodies (Roche, Mannheim, Germany). Corresponding secondary antibodies used for this study were: Cy2 anti-mouse, texas red anti-mouse, alexa647 anti-rabbit, texas red anti-rabbit, Cy2 anti-guinea pig and texas red anti-goat; all obtained from Dianova (Hamburg, Germany) and used as suggested by the manufacturer.

### Immunohistochemistry

Double-label immunofluorescence analyses were carried out on paraffin sections of testis or ovary tissue (3–7 µm) as described in [13], [27]. Paraffin sections were prepared for immunofluorescence by first removing the paraffin by two consecutive incubations of 10 min each in Roti-Histol (Carl Roth, Karlsruhe, Germany). Then the tissue sections were rehydrated in a decreasing ethanol series. Subsequently, antigen retrieval was conducted by incubating the slides in antigen unmasking solution (Vector laboratories, Burlingame, CA) at 125°C and 1.5 bar for 7–20 min. After permeabilization of the tissue in PBS containing 0.1% Triton X-100 for 10 min and washing in PBS, slides were blocked for 30 min in blocking solution (5% milk, 5% FCS, 1 mM PMSF; pH 7.4 in PBS). After incubation with the first primary antibody either for 2 hrs at room temperature or overnight at 4°C, slides were washed in PBS and again blocked in blocking solution before incubating the samples with the second primary antibody for another 2 hrs at room temperature. Following two washing steps (10 min each) in PBS and reblocking for 30 min in blocking solution slides were incubated with the appropriate secondary antibodies. DNA was counterstained using Hoechst 33258 (Sigma-Aldrich, Munich, Germany).

### Telomere fluorescence in-situ hybridization (TeloFISH)

To label telomeres and selected proteins simultaneously, we combined telomere fluorescence in situ hybridization (TeloFISH) with immunofluorescence protocols on paraffin sections as described previously [13]. Paraffin sections were rehydrated and antigen retrieval was conducted as described above. Prior to TeloFISH, cells were permeabilized with PBS/0.1% Triton X-100 for 10 min. After rinsing in 2× SSC (0.3M NaCl, 0.03M Na-citrate; pH 7.4) cells were denatured at 95°C for 20 min in 40 µl of hybridization solution (30% formamide, 10% dextrane sulphate, 250 µg/ml *E. coli* DNA in 2× SSC) supplemented with 10 pmol digoxigenin-labeled (TTAGGG)_7_/(CCCTAA)_7_ oligomeres. Hybridization was performed at 37°C overnight in a humid chamber. Slides were washed two times in 2× SSC at 37°C for 10 min each and blocked with 0.5% blocking-reagent (Roche, Mannheim, Germany) in TBS (150 mM NaCl, 10 mM Tris/HCl; pH 7.4). Samples were incubated with mouse anti-digoxigenin antibodies (Roche, Mannheim, Germany) according to the manufacturer's protocol and bound antibodies detected with Cy2-conjugated anti-mouse secondary antibodies. Following the TeloFISH procedure, samples were prepared for immunofluorescence by blocking with PBT (0.15% BSA, 0.1% Tween 20 in PBS, pH 7.4). Slides were incubated with the first primary antibody overnight, washed two times in PBS for 10 min each and incubated with the corresponding secondary antibody as described above. Finally, slides were again washed in PBS before incubating with the second primary antibody. After repeated washing in PBS samples once again were exposed to the corresponding secondary antibodies. DNA was counterstained using Hoechst 33258 (Sigma-Aldrich, Munich, Germany).

### Electron microscopy

For electron microscopy, fresh tissue from testis and ovary was prepared as described in [22]. The tissues were fixed in 2.5% buffered glutaraldehyde solution (2.5% glutaraldehyde, 50 mM KCl, 2.5 mM MgCl, 50 mM cacodylate; pH 7.2) for 45 min and washed in cacodylate buffer (50 mM cacodylate, pH 7.2). This was followed by incubation in 2% osmium tetroxide in 50 mM cacodylate at 0°C. The samples were then washed several times in water at 4°C and contrasted using 0.5% uranyl acetate in water at 4°C overnight. Subsequently, the tissues were dehydrated in an increasing ethanol series and incubated three times in propylene oxide for 30 min. Finally, the samples were embedded in epon for ultrathin sectioning.

### Microscopy and image analysis

Fluorescence images were acquired using a confocal laser scanning microscope (Leica TCS-SP2; Leica, Mannheim, Germany) equipped with a 63x/1.40 HCX PL APO lbd.BL oil-immersion objective. Images shown are pseudo colored by the Leica TCS-SP2 confocal software and are calculated maximum projections of sequential single sections. These were processed using Adobe Photoshop (Adobe Systems). 3D reconstructions, as well as analysis and quantification of telomere attachment and clustering were conducted using the ImageJ software (version 1.42q; http://rsbweb.nih.gov/ij).

## Supporting Information

Figure S1 Meiotic telomere attachment in early leptotene and zygotene spermatocytes. Representative spermatocytes in paraffin sections of *Sun1^+/+(Δex10-11)^* and *Sun1^−/−(Δex10-11)^* 12 dpp testis tissue labeled by TeloFISH in combination with anti-lamin B and anti-SYCP3 antibodies. In early leptotene spermatocytes full telomere attachment is not yet reached, even in the wildtype. Some internal telomere signals are still detectable in the wildtype spermatocytes, probably due to the early meiotic stage. Comparable stages of spermatocytes from knockout mice also show reduced telomere attachment compared to later meiotic stages (see Figure 1D). During zygotene, as judged by SYCP3 staining, all telomeres in wildtype spermatocytes are attached to the NE as no internal telomere signals are detected anymore. In spermatocytes from knockout tissue of comparable stages, internal telomere signals are still visible, yet more telomeres are attached than in earlier meiotic stages (see Figure 1D′). Scale bars 5 µm.
(TIF)

Video S1 3-dimensional reconstructions of entire spermatocyte nuclei showing clustered and non-clustered telomere patterns. Representative spermatocytes of paraffin testis sections of *Sun1^(Δex10-11)^* wildtype and knockout males labeled by KASH5 (green) and SYCP3 (red). Non-clustered KASH5 foci, marking telomeres attached to the NE, in pachytene cells and clustered KASH5 foci representing the earlier bouquet stage can clearly be observed in wildtype spermatocytes. In SUN1 deficient spermatocytes, non-clustered and clustered patterns of KASH5 foci can also be observed. Here, clustered KASH5 foci also represent bouquet-like formations of successfully attached telomeres. Scale bars 5 µm.
(AVI)
