# Supplementary figures and images for: Analysis of Meiosis in SUN1 Deficient Mice Reveals a Distinct Role of SUN2 in Mammalian Meiotic LINC Complex Formation and Function
Source: PLoS Genet. 2014 Feb 27;10(2):e1004099. doi: 10.1371/journal.pgen.1004099 (PMC3937131; doi:10.1371/journal.pgen.1004099)

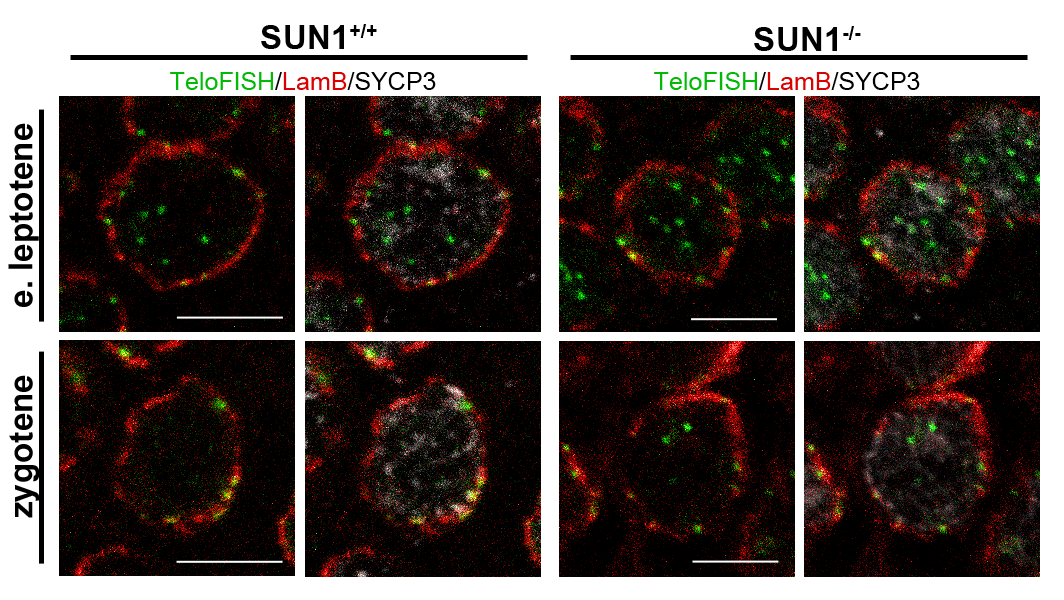

Supplement: Figure S1 — Meiotic telomere attachment in early leptotene and zygotene spermatocytes. Representative spermatocytes in paraffin sections of Sun1+/+(Δex10-11) and Sun1−/−(Δex10-11) 12 dpp testis tissue labeled by TeloFISH in combination with anti-lamin B and anti-SYCP3 antibodies. In early leptotene spermatocytes full telomere attachment is not yet reached, even in the wildtype. Some internal telomere signals are still detectable in the wildtype spermatocytes, probably due to the early meiotic stage. Comparable stages of spermatocytes from knockout mice also show reduced telomere attachment compared to later meiotic stages (see Figure 1D). During zygotene, as judged by SYCP3 staining, all telomeres in wildtype spermatocytes are attached to the NE as no internal telomere signals are detected anymore. In spermatocytes from knockout tissue of comparable stages, internal telomere signals are still visible, yet more telomeres are attached than in earlier meiotic stages (see Figure 1D′). Scale bars 5 µm. (TIF) [file pgen.1004099.s001.tif]
